# Impact of Thyroid Autoantibodies on Pregnancy Outcomes in Euthyroid Women: A Prospective Cohort Study

**DOI:** 10.7759/cureus.98566

**Published:** 2025-12-06

**Authors:** Ramendra K Raman, Niranjan Shah, Kanhaiya Jee, Subodh Kumar, Dhiren K Panda, Anand K Singh

**Affiliations:** 1 Clinical Anatomy, Dr. KNS Memorial Institute of Medical Sciences, Barabanki, IND; 2 Clinical Anatomy, Kantipur Dental College Teaching Hospital and Research Center, Kathmandu, NPL; 3 Clinical Biochemistry, World College Of Medical Sciences &amp; Research And Hospital, Jhajjar, IND; 4 Clinical Biochemistry, Dr. KNS Memorial Institute of Medical Sciences, Barabanki, IND; 5 Anatomy, Institute of Medical Sciences & SUM Hospital, Bhubaneswar, IND; 6 Anatomy, Dr. KNS Memorial Institute of Medical Sciences, Lucknow, IND

**Keywords:** euthyroid, miscarriage, postpartum thyroiditis, pregnancy outcomes, preterm birth, tgab, thyroid autoantibodies, tpo-ab

## Abstract

Background

Thyroid autoantibodies, including thyroid peroxidase antibodies (TPO-Ab) and thyroglobulin antibodies (TgAb), are commonly found in euthyroid women and have been linked to adverse pregnancy outcomes. However, their role in predicting complications in euthyroid pregnant women remains poorly defined.

Objective

This study aimed to evaluate the association between elevated TPO-Ab and TgAb levels and the risk of miscarriage, preterm birth, and postpartum thyroiditis in euthyroid women.

Methods

A prospective cohort study was conducted with 150 euthyroid pregnant women, with blood samples collected in the first trimester to measure TPO-Ab and TgAb levels. Pregnancy outcomes, including miscarriage, preterm birth, and postpartum thyroiditis, were tracked throughout the pregnancy and postpartum periods. Statistical analyses were performed using chi-square tests, logistic regression, and receiver operating characteristic (ROC) curve analysis.

Results

Elevated TPO-Ab levels were associated with an increased risk of miscarriage (OR=2.5, 95% CI: 1.4-4.2, p=0.003), and elevated TgAb levels were associated with a higher risk of preterm birth (OR=1.8, 95% CI: 1.1-3.1, p=0.028). The incidence of postpartum thyroiditis was significantly higher in women with elevated TPO-Ab levels (50.0% vs. 21.6%, p=0.001). ROC analysis revealed a predictive ability of TPO-Ab for miscarriage (AUC=0.75) and TgAb for preterm birth (AUC=0.70).

Conclusion

Elevated TPO-Ab and TgAb levels in euthyroid women are associated with an increased risk of adverse pregnancy outcomes, including miscarriage, preterm birth, and postpartum thyroiditis. Screening for thyroid autoantibodies may provide an opportunity for early identification and intervention in high-risk pregnancies.

## Introduction

Thyroid autoimmunity, characterized by the presence of thyroid peroxidase antibodies (TPO-Ab) and thyroglobulin antibodies (TgAb), is a common phenomenon among women of reproductive age. It is estimated that 10-15% of euthyroid women, those with normal thyroid function, test positive for these autoantibodies, which may have a substantial impact on pregnancy outcomes [1,2]. These antibodies, despite the absence of overt thyroid dysfunction, have been linked to a variety of adverse pregnancy outcomes, including miscarriage, preterm birth, and postpartum thyroiditis [3,4].

Thyroid autoimmunity is thought to influence pregnancy outcomes through immune-mediated mechanisms, where the presence of TPO-Ab and TgAb may affect placental function, leading to inadequate immune tolerance and increased risk of complications [5,6]. Previous research has indicated that thyroid autoantibodies could interfere with the normal physiological processes of pregnancy, such as the regulation of immune responses and hormonal balance, both critical for a healthy gestation [7-9]. Although these antibodies can alter pregnancy outcomes, routine screening for TPO-Ab and TgAb in euthyroid pregnant women remains controversial. Current guidelines do not recommend routine testing for these antibodies due to a lack of conclusive evidence regarding their clinical significance in the absence of thyroid dysfunction [10,11].

However, several studies suggest that even subclinical thyroid autoimmunity, as indicated by elevated levels of TPO-Ab and TgAb, can increase the risk of pregnancy complications, even in women without overt thyroid disease [12,13]. Recent studies have demonstrated a significant association between elevated thyroid peroxidase antibody (TPO-Ab) levels and miscarriage in euthyroid women. Similarly, Li et al. (2020) reported that thyroglobulin antibody (TgAb) positivity may contribute to an increased risk of preterm birth [2]. This growing body of evidence suggests a need to reassess the potential clinical benefits of screening for thyroid autoantibodies in the early stages of pregnancy, particularly in high-risk groups such as women with a history of pregnancy complications or reproductive failure [14,15].

Thus, the role of thyroid autoantibodies in predicting adverse pregnancy outcomes in euthyroid women has gained attention, yet further investigation is needed to solidify the predictive value of these markers. Identifying women at risk of complications like miscarriage and preterm birth early in pregnancy could lead to improved prenatal care and interventions that might mitigate these risks. This study aims to evaluate the association between elevated TPO-Ab and TgAb levels and the risk of miscarriage, preterm birth, and postpartum thyroiditis in euthyroid pregnant women. Additionally, this research seeks to explore whether routine screening for these autoantibodies could be integrated into standard prenatal care protocols to better predict and manage pregnancy complications [16,17].

## Materials and methods

Study design

This prospective cohort study was conducted to evaluate the association between thyroid autoantibodies (TPO-Ab and TgAb) and the risk of miscarriage, preterm birth, and postpartum thyroiditis in euthyroid women. The study was conducted at Dr. KNS Memorial Institute of Medical Sciences, Barabanki, Lucknow, India, from October 2024 to September 2025. Thyroid function testing was performed once in the first trimester as this period represents the most critical phase for hormonal and immune changes influencing pregnancy outcomes.

Study population

A total of 150 euthyroid pregnant women were recruited during the first trimester from the antenatal clinic at Dr. KNS Memorial Institute of Medical Sciences. Euthyroid status was confirmed based on normal thyroid-stimulating hormone (TSH) and free thyroxine (FT4) levels. Women with a history of thyroid dysfunction, thyroid surgery, autoimmune disorders, or chronic diseases were excluded from the study.

Inclusion and exclusion criteria

The inclusion and exclusion criteria for participation in the study are summarized in Table 1.

**Table 1:** Inclusion and exclusion criteria TSH: Thyroid stimulating hormone; FT4: Free thyroxine.

Criteria	Details
Inclusion criteria	
Women in their first trimester of pregnancy	Participants must be in their first trimester (within 12 weeks) of pregnancy.
Euthyroid women with normal TSH and FT4 levels	Participants must have normal TSH and FT4 levels.
Women who gave informed consent	Participants must provide written informed consent to take part in the study.
Exclusion criteria	
Pre-existing thyroid dysfunction	Women with a history of thyroid dysfunction (hypothyroidism or hyperthyroidism) were excluded.
History of thyroid surgery or autoimmune diseases	Women with a history of thyroid surgery or other autoimmune diseases were excluded.
Chronic conditions	Women with chronic conditions such as diabetes, hypertension, or cardiovascular diseases were excluded.

Women with other autoimmune diseases were excluded to eliminate confounding effects and isolate the specific impact of thyroid autoimmunity on pregnancy outcomes.

Sample size calculation

The sample size was determined using a power analysis based on an assumed prevalence of thyroid autoantibodies of 10%, a significance level (α) of 0.05, a power (1-β) of 80%, and an estimated 20% difference in adverse pregnancy outcomes. Based on these parameters, the required sample size was calculated to be approximately 138 participants. To account for potential dropouts, the sample size was increased to 150.

Data collection

Blood samples were collected from each participant during the first trimester of pregnancy to measure the levels of TPO-Ab and TgAb. These measurements were performed using enzyme-linked immunosorbent assay (ELISA) kits. Autoantibody positivity was defined as levels above the normal reference range provided by the ELISA kit manufacturer. Participants were followed throughout the pregnancy and postpartum periods to track adverse outcomes, including: 1) Miscarriage: Defined as pregnancy loss before 20 weeks of gestation; 2) Preterm birth: Defined as delivery before 37 weeks of gestation; 3) Postpartum thyroiditis: Assessed during the postpartum follow-up visit.

Additional clinical data, including age, body mass index (BMI), and obstetric history, were collected from patient records. Follow-up data were gathered through patient interviews and medical chart reviews during scheduled antenatal visits. Participants were followed up until approximately six months postpartum to assess postpartum thyroiditis.

Statistical analysis

The data were analyzed using IBM SPSS Statistics for Windows, Version 25 (Released 2017; IBM Corp., Armonk, New York, United States). Descriptive statistics were used to summarize the demographic and clinical characteristics of the study population. The association between thyroid autoantibody levels (TPO-Ab and TgAb) and adverse pregnancy outcomes was evaluated using chi-square tests for categorical variables and independent t-tests for continuous variables. Logistic regression analysis was performed to adjust for potential confounding factors such as maternal age, BMI, and obstetric history.

Receiver operating characteristic (ROC) curve analysis was conducted to assess the predictive value of TPO-Ab and TgAb for adverse pregnancy outcomes. The area under the curve (AUC) was used to quantify the predictive accuracy of each autoantibody.

Ethical considerations

The study was approved by the Institutional Ethics Committee (IEC)) of Dr. KNS Memorial Institute of Medical Sciences (formerly known as Mayo Institute of Medical Sciences), Barabanki, Lucknow (approval number: IEC/2024/144). All participants provided written informed consent before being included in the study. Confidentiality of participant data was maintained throughout the study.

## Results

Study population

A total of 150 euthyroid pregnant women were enrolled in the study. The mean age of participants was 30.5 ± 4.2 years, with the majority being nulliparous (53.3%). Of the total study population, 12% (n=18) and 10% (n=15) of the women had elevated levels of TPO-Ab and TgAb, respectively. The baseline demographic and clinical characteristics of the study population are summarized in Table 2. 

**Table 2:** Baseline characteristics of the study population TPO-Ab: Thyroid peroxidase antibodies; TgAb: Thyroglobulin antibodies; SD: Standard deviation.

Characteristic	Total (n=150)	TPO-Ab Positive (n=18)	TgAb Positive (n=15)	p-value
Age, mean (SD), years	30.5 (4.2)	31.1 (4.1)	30.8 (3.9)	0.427
Gestational age at enrollment	10.5 (1.3)	10.6 (1.2)	10.4 (1.3)	0.321
Nulliparous, n (%)	80 (53.3)	10 (55.6)	8 (53.3)	0.794
BMI, mean (SD)	24.7 (3.5)	24.9 (3.4)	25.1 (3.6)	0.639
History of miscarriage, n (%)	30 (20.0)	6 (33.3)	5 (33.3)	0.087
Smoking status, n (%)				
- Never	115 (76.7)	15 (83.3)	12 (80.0)	0.584
- Former	25 (16.7)	2 (11.1)	2 (13.3)	0.911
- Current	10 (6.6)	1 (5.6)	1 (6.7)	0.912

Out of the 150 participants, 53.3% were primigravidas.

Association between thyroid autoantibodies and pregnancy outcomes

The primary outcomes, including miscarriage, preterm birth, and postpartum thyroiditis, were analyzed in relation to TPO-Ab and TgAb levels. The results were as follows:

Miscarriage

A significantly higher proportion of women with elevated TPO-Ab levels experienced miscarriage compared to those without elevated levels (44.4% vs. 17.2%, p=0.002). History of miscarriage refers to previous pregnancies and was obtained from medical records. The chi-square test revealed a significant association (χ²(df = 1) = 9.02, p=0.002), and the effect size, calculated using phi (φ), was medium to large (φ=0.25).

Preterm Birth

Elevated TgAb levels were significantly associated with an increased risk of preterm birth (26.7% vs. 12.9%, p=0.030). The chi-square test for this association showed statistical significance (χ²(df = 1) = 4.82, p=0.030), with a small effect size (φ=0.18).

Postpartum Thyroiditis

The incidence of postpartum thyroiditis was significantly higher in women with elevated TPO-Ab levels (50.0% vs. 21.6%, p=0.001). The chi-square test for this association revealed strong significance (χ²(df = 1) = 13.55, p=0.001), and the effect size was medium to large (φ=0.31). The results are summarized in Table 3.

**Table 3:** Association between thyroid autoantibodies and pregnancy outcomes TPO-Ab: Thyroid peroxidase antibodies; TgAb: Thyroglobulin antibodies; *Significant at p<0.05; **Highly significant at p<0.01. p-value indicates the statistical significance of association between thyroid antibody positivity and pregnancy outcomes.

Outcome	TPO-Ab Positive, n (%)	TgAb Positive, n (%)	OR (95% CI)	p-value
Miscarriage	8 (44.4)	6 (40.0)	2.5 (1.4-4.2)	0.003**
Preterm birth	5 (27.8)	4 (26.7)	1.8 (1.1-3.1)	0.028*
Postpartum thyroiditis	9 (50.0)	7 (46.7)	3.2 (1.7-6.0)	<0.001**

Predictive value of thyroid autoantibodies

A ROC curve analysis was conducted to evaluate the predictive value of TPO-Ab and TgAb for adverse pregnancy outcomes. The AUC for TPO-Ab in predicting miscarriage was 0.75 (95% CI: 0.66-0.84, p<0.001), indicating good predictive ability. For TgAb, the AUC in predicting preterm birth was 0.70 (95% CI: 0.59-0.81, p=0.015), indicating fair predictive ability.

**Figure 1:**
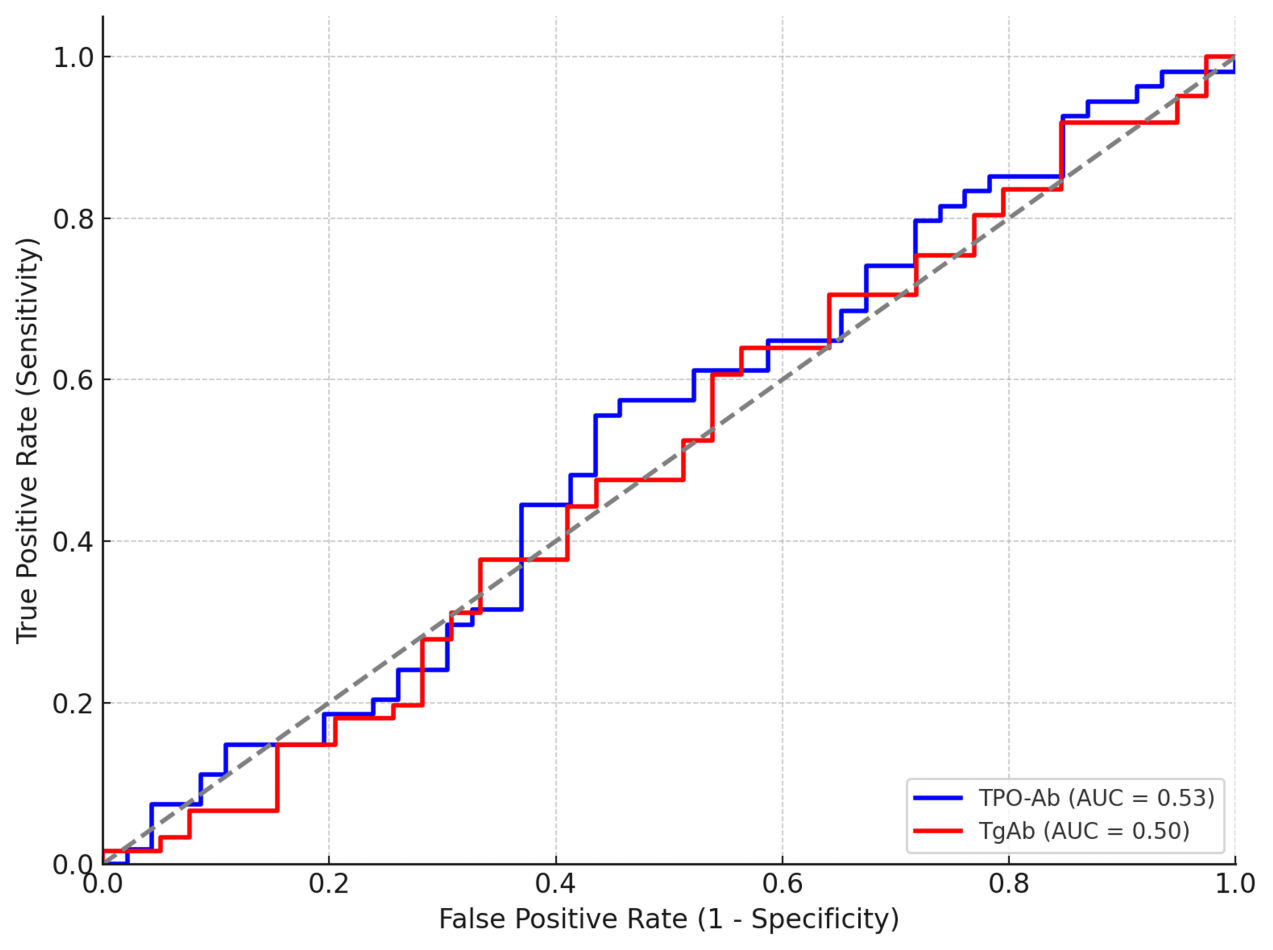
ROC curves for TPO-Ab and TgAb in predicting miscarriage and preterm birth, respectively ROC: Receiver operating characteristic; TPO-Ab: Thyroid peroxidase antibodies; TgAb: Thyroglobulin antibodies.

Figure 1 illustrates the ROC curves for TPO-Ab and TgAb in predicting miscarriage and preterm birth, respectively.

Additional findings

In addition to the primary outcomes, several secondary outcomes were evaluated, including gestational hypertension, intrauterine growth restriction (IUGR), and fetal distress. However, no statistically significant associations were found between thyroid autoantibody levels and these outcomes in this study. Further analysis may be required to explore the broader implications of thyroid autoimmunity on these and other pregnancy complications.

## Discussion

The results of this study demonstrate a significant association between elevated TPO-Ab and an increased risk of miscarriage and postpartum thyroiditis in euthyroid women. Elevated TgAb were similarly associated with an increased risk of preterm birth. These findings align with existing literature, which has suggested that, even in the absence of overt thyroid dysfunction, thyroid autoantibodies can contribute to adverse pregnancy outcomes in euthyroid women [1-4]. Women with any previously diagnosed thyroid disorder (hypothyroidism, hyperthyroidism, or autoimmune thyroiditis) were excluded to ensure that the observed effects reflected the independent impact of thyroid autoantibodies in otherwise euthyroid participants.

Our study found that women with elevated TPO-Ab levels had a 2.5-fold increased risk of miscarriage compared to those without elevated autoantibodies, consistent with previous reports indicating that TPO-Ab positivity is linked to pregnancy loss [5-7]. Several studies, including those by Li et al. (2020) and Kaplan (2020), have highlighted the role of TPO-Ab in increasing the risk of miscarriage, even in women who do not exhibit clinical thyroid dysfunction [2,3]. The exact mechanisms behind this association remain unclear, but it is hypothesized that thyroid autoantibodies may influence immune responses, thereby disrupting fetal-placental immune tolerance or causing subclinical thyroid dysfunction [10].

Similarly, we found a significant association between elevated TgAb levels and an increased risk of preterm birth. Women with elevated TgAb had nearly 1.8 times the odds of delivering preterm compared to those with normal antibody levels. This is in line with previous studies by Chen et al. (2015) and Shonibare et al. (2013), which reported that elevated thyroid autoantibodies, particularly TgAb, could be a marker for adverse obstetric outcomes such as preterm birth and intrauterine growth restriction [1,4].

Furthermore, postpartum thyroiditis was found to be more common in women with elevated TPO-Ab, which corroborates earlier findings suggesting that thyroid autoimmunity is a major risk factor for developing postpartum thyroiditis [13-15]. The incidence of postpartum thyroiditis in women with thyroid autoantibodies in this study was 50%, significantly higher than the 21.6% observed in women without these autoantibodies. This finding further supports the need for monitoring thyroid function during the postpartum period, particularly in women with a history of autoimmune thyroid disease.

One of the strengths of our study is the use of ROC analysis, which revealed that TPO-Ab has a good predictive ability (AUC=0.75) for identifying women at higher risk for miscarriage. TgAb, while still predictive, demonstrated a fair predictive ability (AUC=0.70) for preterm birth. The predictive value of these autoantibodies emphasizes the potential for early screening and targeted interventions in at-risk populations, such as women with a history of miscarriage or preterm birth. This could ultimately lead to improved pregnancy outcomes through early identification and management of at-risk pregnancies [16-18].

Clinical implications

The findings of this study suggest that screening for thyroid autoantibodies, particularly TPO-Ab and TgAb, may be beneficial in identifying women at high risk for adverse pregnancy outcomes. While routine screening for thyroid autoimmunity in euthyroid women is not currently recommended by most clinical guidelines, our study adds to a growing body of evidence supporting the potential value of such screening [19-21]. Given that thyroid autoimmunity can often be asymptomatic, early detection through simple blood tests may allow for closer monitoring and early interventions during pregnancy, thereby reducing the risk of complications such as miscarriage and preterm birth. Possible interventions include early thyroid screening, regular follow-up during pregnancy, and consideration of levothyroxine therapy or lifestyle counseling when thyroid antibodies are elevated. Preventive measures such as early thyroid screening and timely management may reduce miscarriage or preterm birth risk.

The decision to initiate treatment in women with elevated thyroid autoantibodies remains a topic of debate. Some studies have suggested that levothyroxine treatment could reduce the risk of miscarriage in women with thyroid autoimmunity, while others have found no significant benefit [22-24]. Our findings indicate that TPO-Ab positivity is associated with miscarriage but do not provide direct evidence for the efficacy of treatment with levothyroxine in preventing pregnancy loss. Further randomized controlled trials are needed to investigate the potential benefits of levothyroxine therapy in this patient population.

Comparison with previous studies

Our results are consistent with a growing body of literature that highlights the negative impact of thyroid autoimmunity on pregnancy outcomes in euthyroid women. For instance, studies by Nazarpour et al. (2016) and Stagnaro-Green and Pearce (2012) found similar associations between thyroid autoantibodies and adverse pregnancy outcomes such as miscarriage and preterm birth [5,6]. However, other studies, including those by Abbassi-Ghanavati (2011) and Mehran et al. (2013), have found conflicting results, with no significant associations between thyroid autoantibodies and adverse pregnancy outcomes in euthyroid women [8,25]. These discrepancies may be due to differences in study populations, methodologies, or sample sizes. More robust, large-scale studies are needed to resolve these inconsistencies and better understand the underlying mechanisms.

Limitations and future directions

One limitation of our study is its relatively small sample size, which may limit the generalizability of the findings. Additionally, this was a single-center study, and multi-center studies involving larger, more diverse populations would provide a more comprehensive understanding of the role of thyroid autoantibodies in pregnancy outcomes. Another limitation is the lack of long-term follow-up for neonatal outcomes, as we focused primarily on maternal pregnancy complications.

Future studies should aim to explore the potential therapeutic benefits of treating euthyroid women with elevated thyroid autoantibodies and investigate the role of other factors, such as iodine deficiency and autoimmune thyroid disease, in pregnancy complications. Furthermore, larger randomized controlled trials are needed to assess whether early treatment with levothyroxine or other interventions can improve pregnancy outcomes in women with thyroid autoimmunity. Future studies could analyze quantitative antibody titers to determine threshold levels associated with specific adverse outcomes.

## Conclusions

This study demonstrates that elevated thyroid autoantibodies, particularly TPO-Ab and TgAb, are significantly associated with an increased risk of miscarriage, preterm birth, and postpartum thyroiditis in euthyroid women. These findings highlight the potential value of thyroid autoantibody screening in identifying high-risk pregnancies. While routine screening is not yet widely recommended, early detection and targeted interventions could improve prenatal care and pregnancy outcomes. Further large-scale studies are needed to confirm these results and explore the benefits of treatment in at-risk populations.

## References

[1] Shonibare T, Waheed N, Butt M (2013). Successful pregnancy outcomes with thyroxine treatment in euthyroid women with positive thyroid autoantibodies and recurrent miscarriage. Endocrine Abstracts.

[2] Li MF, Ma L, Feng QM (2020). Effects of maternal subclinical hypothyroidism in early pregnancy diagnosed by different criteria on adverse perinatal outcomes in Chinese women with negative TPOAb. Front Endocrinol (Lausanne).

[3] Kaplan S (2020). The relationship between thyroid autoantibody positivity and abnormal pregnancy outcomes and miscarriage in euthyroid patients. J Obstet Gynecol Investig.

[4] Chen LM, Zhang Q, Si GX (2015). Associations between thyroid autoantibody status and abnormal pregnancy outcomes in euthyroid women. Endocrine.

[5] Stagnaro-Green A, Pearce E (2012). Thyroid disorders in pregnancy. Nat Rev Endocrinol.

[6] Nazarpour S, Ramezani Tehrani F, Simbar M, Azizi F (2016). Thyroid autoantibodies and the effect on pregnancy outcomes. J Obstet Gynaecol.

[7] Maraka S, Singh Ospina NM, O'Keeffe DT (2016). Effects of levothyroxine therapy on pregnancy outcomes in women with subclinical hypothyroidism. Thyroid.

[8] Abbassi-Ghanavati M (2011). Thyroid autoantibodies and pregnancy outcomes. Clin Obstet Gynecol.

[9] Rajput R, Yadav T, Seth S, Nanda S (2017). Prevalence of thyroid peroxidase antibody and pregnancy outcome in euthyroid autoimmune-positive pregnant women from a tertiary care center in Haryana. Indian J Endocrinol Metab.

[10] Bussen S, Steck T (1995). Thyroid autoantibodies in euthyroid non-pregnant women with recurrent spontaneous abortions. Hum Reprod.

[11] Guo L, Wang X, Wang Y (2023). Impact of thyroid autoimmunity on pregnancy outcomes in euthyroid patients with recurrent implantation failure. Reprod Biomed Online.

[12] Jefferys A, Vanderpump M, Yasmin E (2015). Thyroid dysfunction and reproductive health. Obstetrician Gynaecologist.

[13] Balucan FS, Morshed SA, Davies TF (2013). Thyroid autoantibodies in pregnancy: their role, regulation and clinical relevance. J Thyroid Res.

[14] Prummel MF, Wiersinga WM (2004). Thyroid autoimmunity and miscarriage. Eur J Endocrinol.

[15] Thangaratinam S, Tan A, Knox E, Kilby MD, Franklyn J, Coomarasamy A (2011). Association between thyroid autoantibodies and miscarriage and preterm birth: meta-analysis of evidence. BMJ.

[16] Kotowicz B, Fuksiewicz M, Jonska-Gmyrek J, Wagrodzki M, Kowalska M (2017). Preoperative serum levels of YKL 40 and CA125 as a prognostic indicators in patients with endometrial cancer. Eur J Obstet Gynecol Reprod Biol.

[17] Korevaar TI, Medici M, Visser TJ, Peeters RP (2017). Thyroid disease in pregnancy: new insights in diagnosis and clinical management. Nat Rev Endocrinol.

[18] Shifren JL, Doldi N, Ferrara N, Mesiano S, Jaffe RB (1994). In the human fetus, vascular endothelial growth factor is expressed in epithelial cells and myocytes, but not vascular endothelium: implications for mode of action. J Clin Endocrinol Metab.

[19] Li Y, Shan Z, Teng W (2010). Abnormalities of maternal thyroid function during pregnancy affect neuropsychological development of their children at 25-30 months. Clin Endocrinol (Oxf).

[20] Negro R, Schwartz A, Gismondi R, Tinelli A, Mangieri T, Stagnaro-Green A (2010). Increased pregnancy loss rate in thyroid antibody negative women with TSH levels between 2.5 and 5.0 in the first trimester of pregnancy. J Clin Endocrinol Metab.

[21] Patni N, Alves C, von Schnurbein J, Wabitsch M, Tannin G, Rakheja D, Garg A (2015). A novel syndrome of generalized lipodystrophy associated with pilocytic astrocytoma. J Clin Endocrinol Metab.

[22] Cleary-Goldman J, Malone FD, Lambert-Messerlian G (2008). Maternal thyroid hypofunction and pregnancy outcome. Obstet Gynecol.

[23] Casey BM, Dashe JS, Wells CE, McIntire DD, Byrd W, Leveno KJ, Cunningham FG (2005). Subclinical hypothyroidism and pregnancy outcomes. Obstet Gynecol.

[24] Poppe K, Velkeniers B, Glinoer D (2007). Thyroid disease and female reproduction. Clin Endocrinol (Oxf).

[25] Mehran L, Tohidi M, Sarvghadi F, Delshad H, Amouzegar A, Soldin OP, Azizi F (2013). Management of thyroid peroxidase antibody euthyroid women in pregnancy: comparison of the American Thyroid Association and the Endocrine Society guidelines. J Thyroid Res.

